# Broad complex rhythm with a salty taste

**DOI:** 10.1007/s12471-017-0951-x

**Published:** 2017-01-20

**Authors:** M. Boulaksil, C. L. Meuwese, R. Evertz, M. G. M. Kolff-Kamphuis

**Affiliations:** 1 Department of Cardiology, Jeroen Bosch Hospital, ’s-Hertogenbosch, The Netherlands; 2 Department of Cardiology, Radboud University Medical Center, Nijmegen, The Netherlands; 3 Department of Cardiology, University Medical Center Utrecht, Utrecht, The Netherlands

## Answer

**Fig. 1:**
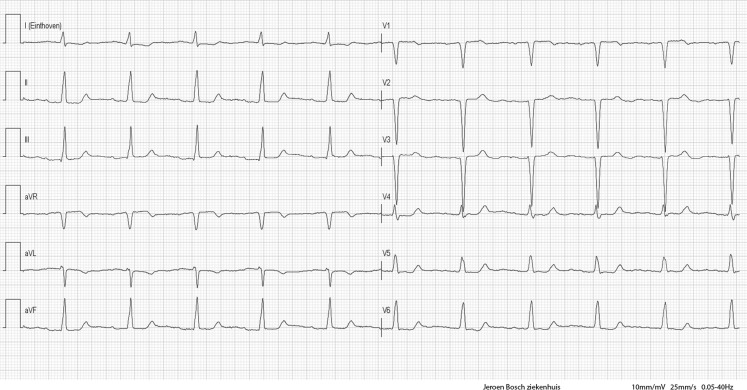
Follow-up ECG

The ECG (Fig. 1 in the question) shows an extremely broad QRS complex rhythm (QRS duration of 220 ms) with a heart rate of 95 beats/min and a left axis deviation. The differential diagnosis of a broad QRS complex rhythm (without tachycardia) is extensive, including bundle branch block (either rate dependent or fixed), hyperkalaemia, pre-excitation, ventricular pacing, (accelerated) idioventricular rhythm, or hypothermia, but also intoxication with a sodium-channel blocking agent (e. g. tricyclic antidepressants or class I antiarrhythmic drugs (Vaughan-Williams classification)).

As our patient had a history of paroxysmal atrial fibrillation, for which she was treated with flecainide, a class Ic antiarrhythmic drug, and no alternative explanation for her broad QRS complex rhythm was found, we concluded she was suffering from flecainide intoxication. Since flecainide is mainly cleared by the renal route, a reduced kidney function due to dehydration likely aggravated the toxic effects of flecainide in our patient.

As the first already abnormal ECG was not recognised at presentation, she was allowed to take her daily dose of flecainide, which further worsened her symptoms and ECG abnormalities (Fig. 2 in the question).

Shortly after admission to the Cardiac Care Unit she developed cardiogenic shock for which low-dose intravenous inotropic agents were initiated. During admission, her clinical condition improved and the ECG became comparable with previous ECGs (Fig. 1).

Certain loss-of-function sodium channel mutations leading to the typical Brugada ECG pattern may also result in less availability of sodium channels. However, in the typical Brugada ECG pattern, the ECG abnormalities are confined to the right precordial leads (ST-segment elevation and RBBB-like pattern) since the myocardium of the right ventricular outflow tract (RVOT) is mainly affected. In our patient, however, sodium channel blockade was not focused at the RVOT level, but was generalised to the whole heart and across the myocardial layers. We hypothesise this is the reason why an LBBB-like pattern is seen instead and no typical ST-segment elevation.

Intoxication with flecainide is associated with a high mortality rate [1]. Treatment of severe flecainide intoxication is hampered by its pharmacokinetics and pharmacodynamics. Furthermore, haemodialysis, peritoneal dialysis, and haemofiltration have not been proven effective. Therefore, supportive treatment remains the cornerstone therapy in severe flecainide intoxication [2, 3].

## Conclusion

Flecainide intoxication.
